# Advancing a systems-level understanding of neurodegeneration: the BrightFocus Alzheimer’s disease research portfolio

**DOI:** 10.1186/s13024-026-00984-8

**Published:** 2026-08-21

**Authors:** Sharyn L. Rossi, Diane E. Bovenkamp

**Affiliations:** BrightFocus Foundation, Clarksburg, MD United States of America

## Abstract

Alzheimer’s disease research is entering a period of rapid acceleration. After decades focused on identifying individual disease-associated proteins and pathways, the field is moving toward a deeper understanding of how these mechanisms interact to influence cellular function, biological systems, and ultimately behavior and cognition. The emphasis is moving from isolated molecular targets to coordinated biological systems. Underlying pathological mechanisms point toward the breakdown of core cellular maintenance systems, including proteostasis, metabolism, and immune regulation. This dysfunction propagates from organelles to cells, from cells to neural circuits, and, ultimately, to cognition and behavior. Moving beyond single-target approaches will depend on understanding these upstream drivers, how they interact, and how sex, genetic background, and accumulated stressors shape when and how these systems fail. Underlying pathological mechanisms point toward the breakdown of core cellular maintenance systems, including proteostasis, metabolism, and immune regulation. This dysfunction propagates from organelles to cells, from cells to neural circuits, and ultimately to cognition and behavior. The BrightFocus Alzheimer’s Disease Research (ADR) program [1] has long operated within this broad framework. Rather than focusing narrowly on single disease targets, the portfolio takes a 360° approach and prioritizes innovative research that reveals how fundamental cellular processes interact across cell types and biological systems to drive neurodegeneration and resilience. This includes supporting early-career investigators and researchers entering the field from other disciplines to advance current hypotheses and define where the field is going next.

**Supplementary Information:**

The online version contains supplementary material available at 10.1186/s13024-026-00984-8.

## Graphical Abstract

**Figure:**
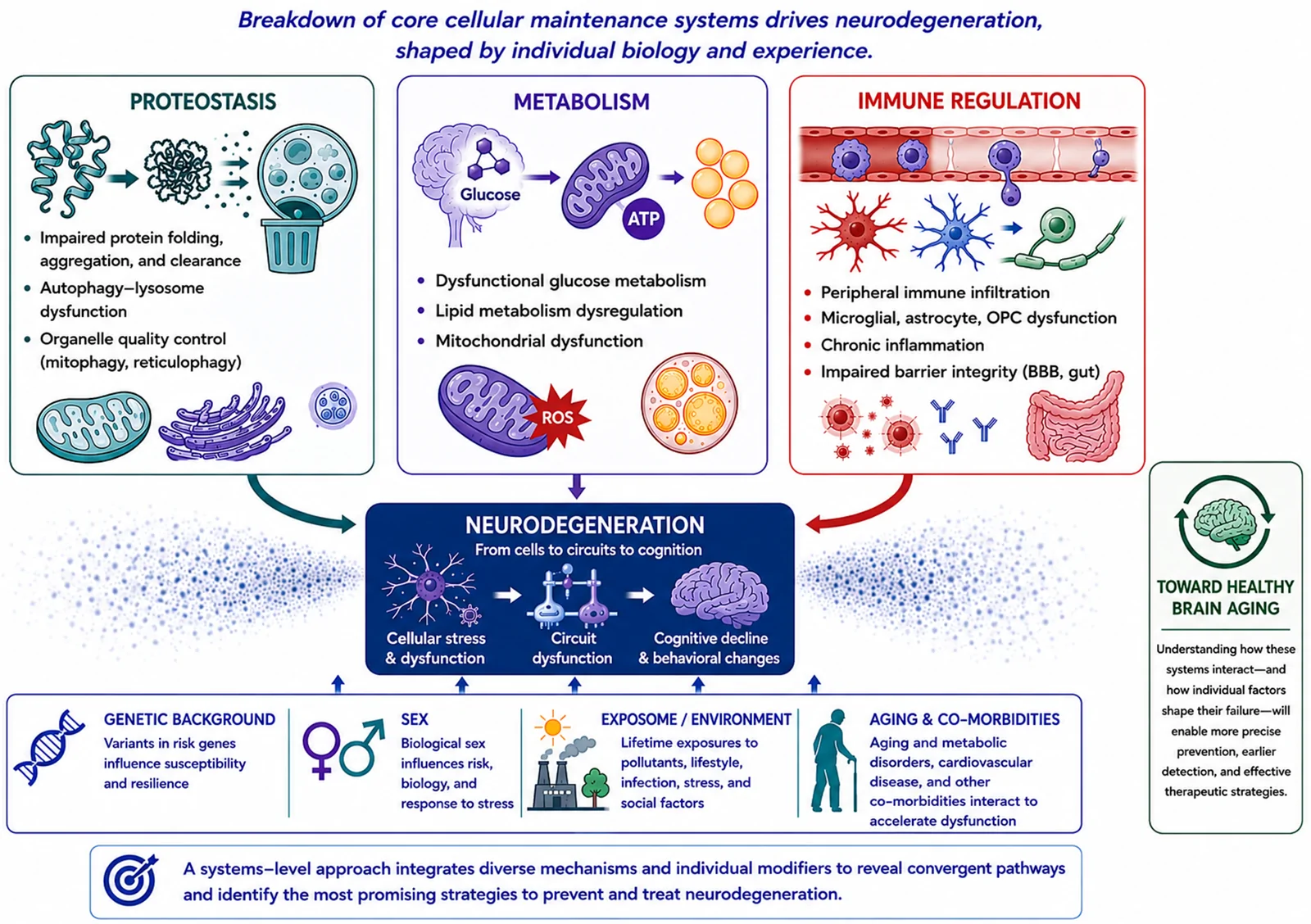

## Introduction

The rapid expansion of multimodal datasets has fundamentally changed the landscape of Alzheimer’s disease research. Advances in genomics, transcriptomics, proteomics, metabolomics, imaging, digital biomarkers, and longitudinal clinical cohorts now provide unprecedented opportunities to examine disease across multiple biological scales. At the same time, these datasets have underscored the remarkable heterogeneity of Alzheimer’s disease, revealing that similar clinical phenotypes can arise through distinct molecular and cellular pathways, while shared biological mechanisms may manifest differently across individuals and populations.

Recent work on centenarians [2], along with findings from the Longitudinal Study on Aging in India - Diagnostic Assessment of Dementia (LASI-DAD) [3], underscores the heterogeneity of Alzheimer’s disease across populations. In these cohorts, amyloid and tau blood biomarkers either do not correlate with cognitive performance or, in the case of LASI-DAD, show an inverse relationship with the Aβ42/40 ratio. These findings highlight the complexity of real-world populations, where genetic ancestry, environmental exposures, vascular and metabolic comorbidities, immune status, and other age-related conditions may influence biomarkers independently of Alzheimer’s disease pathology. Such variability is consistent with the roles that the exposome, population-specific genetic variation, and protective factors play in brain aging, including context-dependent effects of established risk genes such as APOE and TREM2. In contrast, markers such as neurofilament light and GFAP more consistently reflect overall brain health and aging across these cohorts, suggesting that diagnostic approaches centered on overall brain health and functional integrity, rather than solely on disease-specific hallmarks, may prove more reliable and broadly generalizable.

As the volume and complexity of available data grows, the challenge becomes integrating existing evidence into biological frameworks that identify convergent mechanisms, expose critical knowledge gaps, and guide the most informative next experiments. This exposes a limitation in how the disease is studied as many widely used experimental models reproduce selected aspects of pathology through engineered overexpression of specific proteins. While valuable, these systems do not capture the complexity of human disease. Because only a small fraction of Alzheimer’s cases are driven by autosomal dominant mutations, understanding sporadic, late-onset disease requires experimental models that are informed by human pathophysiology and an iterative framework where human populations guide model development. Bringing these diverse perspectives will be essential for accelerating discovery and prioritizing the therapeutic strategies most likely to succeed.

### Proteostasis and cellular maintenance

Proteostatic processes are highly specialized, varying across both cell types and organelles. Structures such as the nucleus, mitochondria, endo/lysosome, and ribosome each require distinct homeostatic programs to support their specialized functions. Autophagy provides an additional layer of quality control within this system by removing aberrant proteins and organelles through pathways such as mitophagy [4] and reticulophagy.

Disruption of proteostasis is a central feature of neurodegenerative disease and BrightFocus-supported research highlights how failures in protein turnover and cellular recycling systems can initiate broader biological consequences. For example, work from Ching-Chieh Chou [5] uses directly converted human neurons to preserve aspects of donor age and genetic background that are typically lost in reprogramming approaches. These induced Alzheimer’s neurons exhibit exacerbated defects in lysosomal repair and protein turnover, contributing to inflammatory signaling and increased vulnerability to cell death.

Disturbances in proteostasis can propagate outward to affect neuronal circuits and larger biological processes. Christopher Morrone’s work demonstrates that autophagic dysfunction within sleep-wake circuitry is linked to early sleep disruption in Alzheimer’s disease [6]. Similarly, Ashish Sharma’s research suggests that defects in ribosomal biology and protein processing can impose circadian stress and promote amyloid pathology [7]. These findings further substantiate proteostatic dysfunction as a driver of systems level dysfunction well before extensive neuronal loss occurs.

### Metabolic homeostasis and bioenergetic stress

Proteostasis is tightly coupled with cellular metabolism. The synthesis, folding, trafficking, and degradation of proteins are energy-intensive processes that rely on efficient glucose metabolism, lipid metabolism, and mitochondrial function. When bioenergetic systems fail, the capacity of cells to maintain proteostasis declines, creating a feedback loop in which metabolic stress accelerates protein dysfunction and cellular damage.

Emerging evidence suggests that Alzheimer’s disease involves profound disturbances in cellular bioenergetics, and BrightFocus-supported work from Shannon Macauley [8–10] highlights how metabolic inflexibility contributes to neurodegeneration. Amyloid and tau pathology drive metabolic reprogramming that alters glucose and lactate metabolism, promotes reactive glial states and neuroinflammation, and contributes to cortical hyperexcitability and sleep disruption. Together, these findings identify metabolic pathways as potential therapeutic targets capable of influencing both disease pathology and physiological function.

### Immune regulation and microglial function

While the brain was once described as immune privileged, it is now more accurately understood as immune specialized, integrating signals from the vasculature, circulating immune factors, and other peripheral inputs. These signals can influence microglial behavior, shaping how the brain responds to stress, injury, and disease. Genetic variants affecting immune and metabolic pathways can alter microglial function in ways that increase disease risk or confer resilience.

BrightFocus-supported work has helped formalize the concept of “border immunity,” describing specialized immune niches at the meninges, choroid plexus, and perivascular spaces where peripheral immune signals interact with central nervous system function. These interfaces represent critical points of regulation rather than passive boundaries [11]. Recent work extends this framework by linking genetic risk and sex to immune regulation at these interfaces. Studies from Sandro da Mesquita and colleagues [12] show that *APOE4* drives sex-specific changes in innate immune signaling that alter meningeal lymphatic function, lipid homeostasis, and neuroinflammation. In this context, APOE functions not only as a regulator of lipid transport within the brain but as part of a broader system governing communication between peripheral immunity and central nervous system physiology. The pleiotropic effects of *APOE* across multiple biological systems and cell types offer important insights into the mechanisms that generate heterogeneity in Alzheimer’s disease.

BrightFocus has supported important work examining how microglial surface receptors respond to pathological signals and genetic perturbation. Restoring or preserving the homeostatic functions of microglia, including debris clearance, trophic support, and neuronal maintenance, remains a major therapeutic priority. Central to this work are the Alzheimer’s disease risk genes *TREM2* and *APOE*, which influence how microglia and other glial cells detect and respond to cellular stress.

These risk genes have become compelling pharmacological targets because they shape the behavior not only of microglia, but also of astrocytes and oligodendrocytes. Exposure of support cells to toxic or pathological signals produces disease-associated cellular states that may be useful for both diagnostic stratification and therapeutic intervention. In this area, Tom Brett and colleagues used biophysical mapping and quantitative reconstruction of molecular interactions to define distinct and biologically meaningful interfaces between TREM2 and several proteins associated with Alzheimer’s disease pathology and inflammation. Their findings indicate that TREM2 behaves less like a receptor for a single ligand and more like a sensor of molecular patterns associated with damage, stress, or cellular debris [10]. Certain *TREM2* variants appear to impair microglial function in sex-dependent ways [11], while others identified in individuals with Down syndrome may confer protection [12]. These findings may also illuminate mechanistic overlap across neurological and neuropsychiatric disorders, including autism [13], schizophrenia, and psychosis, where related immune and glial pathways may be involved.

### Glia, APOE, and oligodendrocytes

Large-scale genetic, transcriptomic, and cellular analyses are pointing toward circuit-level interactions between microglia, astrocytes [13], and, more recently, oligodendrocytes. Particularly in the context of *APOE*, a central regulator of cholesterol trafficking and lipid metabolism, multiple lines of evidence increasingly converge on oligodendrocytes [14]. These myelinating cells provide essential metabolic and structural support to neurons through lipid metabolism, myelin production, and metabolic coupling. Across multiple datasets, some of the most robust cell state changes associated with aging and Alzheimer’s disease occur within oligodendrocyte populations.

This observation is beginning to shift how the field thinks about brain aging itself. Neurons remain central to cognitive function, but neuronal vulnerability is increasingly understood within the context of the glial systems that support them. Oligodendrocytes in particular sit at the intersection of lipid metabolism, energy utilization, and circuit stability and changes in myelin biology play a far larger role in brain aging than previously appreciated.

This has become one of the most important and forward-looking areas in the portfolio. Work from Na Zhao at Mayo Clinic Jacksonville suggests that the protective effects of APOE2 may be linked, at least in part, to preservation or promotion of myelin integrity [15]. That possibility is highly significant for the field. Some of the most promising non-pharmacological interventions may ultimately prove effective in part because they support myelination and broader glial health. One example is gamma-frequency sensory stimulation, including the Cognito flicker approach [16], whose mechanistic underpinnings BrightFocus has continued to support through Annabelle Singer at Georgia Tech [17]. Sex steroids appear to play a key role in regulating myelin biology: clinical work with the neurosteroid allopregnanolone [18], along with evidence that hormonal shifts reshape white matter and brain connectivity, underscores that myelin is a dynamic and highly regulated system whose plasticity may be leveraged in neurodegenerative disease.

### Looking Forward

Neurodegeneration arises from failures in fundamental cellular maintenance systems. Disruptions in proteostasis, metabolism, and immune regulation interact across cell types and biological scales, ultimately influencing neural circuits, resilience, and cognition. How these disruptions manifest and lead to disease depends on underlying genetic architecture and the cumulative environmental and biological exposures experienced throughout aging. Successful interventions will likely need to begin early, be personalized, and combine multiple therapeutic approaches.

The studies highlighted throughout this portfolio illustrate an emerging shift in Alzheimer’s disease research. As lines of investigation continue to converge, the opportunity is not to identify new disease-associated mechanisms, but to integrate existing discoveries into physiological frameworks that explain how interacting biological systems shape distinct trajectories of neurodegeneration. Achieving this goal will require collaboration across laboratories, disciplines, and areas of expertise to connect cross-disease mechanisms spanning molecules, cells, tissues, organ systems, and whole-body physiology.

Reflecting this systems-level view of Alzheimer’s disease, BrightFocus has long recognized and invested in research areas that are now shaping the next phase of the field, including women’s health, oligodendrocyte biology, cross-disease convergence, and whole-body systems integration. For a more detailed analysis of BrightFocus funded contributions to these key areas, see Supplemental Table 1. The BrightFocus portfolio continues to support innovative research in these domains while remaining responsive to new directions emerging across the field.

## Supplementary Information

Below is the link to the electronic supplementary material.

Supplementary Material 1

## Data Availability

No datasets were generated or analysed during the current study.
