# Supplementary material for: Advancing a systems-level understanding of neurodegeneration: the BrightFocus Alzheimer’s disease research portfolio
Source: Mol Neurodegener. 2026 Aug 21;21:50. doi: 10.1186/s13024-026-00984-8 (PMC13495267; doi:10.1186/s13024-026-00984-8)
Supplement: Supplementary file 1 [file 13024_2026_984_MOESM1_ESM.docx]

Table 1: Key Highlights of BrightFocus-Funded Publications Addressing Topics Outlined in *Advancing a Systems-Level Understanding of Neurodegeneration: The BrightFocus Alzheimer’s Disease Research Portfolio* (highly-cited and published within the past 5 years)

A) **Proteostasis and Cellular Maintenance**

| **Title** | **Link To Paper** |
| --- | --- |
| Proteostasis failure exacerbates neuronal circuit dysfunction and sleep impairments in Alzheimer’s disease | <https://link.springer.com/article/10.1186/s13024-023-00617-4> |
| Proteostasis and lysosomal repair deficits in transdifferentiated neurons of Alzheimer's disease | <https://www.nature.com/articles/s41556-025-01623-y> |
| Hippocampal GFAP-positive astrocyte responses to amyloid and tau pathologies | <https://www.sciencedirect.com/science/article/abs/pii/S0889159123000582> |
| Prominent tauopathy and intracellular β-amyloid accumulation triggered by genetic deletion of cathepsin D: implications for Alzheimer disease pathogenesis | <https://link.springer.com/article/10.1186/s13195-024-01443-6> |
| The complexity of extracellular vesicles: Bridging the gap between cellular communication and neuropathology | <https://onlinelibrary.wiley.com/doi/epdf/10.1111/jnc.16108> |
| The role of mitochondrial uncoupling in the regulation of mitostasis after traumatic brain injury | <https://www.sciencedirect.com/science/article/pii/S019701862400007X> |
| CRISPR screen for protein inclusion formation uncovers a role for SRRD in the regulation of intermediate filament dynamics and aggresome assembly | <https://journals.plos.org/plosgenetics/article?id=10.1371/journal.pgen.1011138> |
| Chaperone-dependent and chaperone-independent functions of carboxylate clamp tetratricopeptide repeat (CC-TPR) proteins | <https://linkinghub.elsevier.com/retrieve/pii/S0968000424002561> |
| Stathmin-2 loss leads to neurofilament-dependent axonal collapse driving motor and sensory denervation | <https://www.nature.com/articles/s41593-023-01496-0> |
| The expanding repertoire of ESCRT functions in cell biology and disease | <https://www.nature.com/articles/s41586-025-08950-y> |
| Alzheimer's disease patient-derived high-molecular-weight tau impairs bursting in hippocampal neurons | <https://www.sciencedirect.com/science/article/pii/S0092867425004088> |
| Phosphorylation-driven epichaperome assembly is a regulator of cellular adaptability and proliferation | <https://www.nature.com/articles/s41467-024-53178-5> |
| ATP- sensitive potassium channels alter glycolytic flux to modulate cortical activity and sleep | <https://www.pnas.org/doi/epdf/10.1073/pnas.2416578122> |
| Cationic peptides cause memory loss through endophilin-mediated endocytosis | <https://www.nature.com/articles/s41586-024-08413-w> |

B) **Metabolic Homeostasis and Bioenergetic Stress**

| **Topic** | **Link To Paper** |
| --- | --- |
| Balancing nutrient and energy demand and supply via autophagy | <https://www.sciencedirect.com/science/article/pii/S0960982222007072> |
| Ethanol exposure alters Alzheimer's-related pathology, behavior, and metabolism in APP/PS1 mice | <https://www.sciencedirect.com/science/article/pii/S096999612200359X> |
| Mitophagy in the pathogenesis and management of disease | <https://www.nature.com/articles/s41422-025-01203-7> |
| Wnt5a Promotes Lysosomal Cholesterol Egress and Protects Against Atherosclerosis | <https://www.ahajournals.org/doi/10.1161/CIRCRESAHA.121.318881> |
| Degradative and Non-Degradative Roles of Autophagy Proteins in Metabolism and Metabolic Diseases | <https://www.frontiersin.org/journals/cell-and-developmental-biology/articles/10.3389/fcell.2022.844481/full> |
| Common and divergent pathways in early stages of glutamate and tau-mediated toxicities in neurodegeneration | <https://www.sciencedirect.com/science/article/abs/pii/S0014488624002930?via%3Dihub> |
| Nonobese Male Patients with Alzheimer's Disease Are Vulnerable to Decrease in Plasma Leptin | <https://journals.sagepub.com/doi/abs/10.3233/JAD-220447> |
| Role of dysregulated calcium homeostasis in astrocytes in neurodegenerative disorders | <https://www.nature.com/articles/s41583-026-01032-6> |
| Failure in a population: Tauopathy disrupts homeostatic set-points in emergent dynamics despite stability in the constituent neurons | <https://www.sciencedirect.com/science/article/pii/S0896627324005786> |

C) **Immune Regulation and Microglial Function**

| Topic | Link To Paper |
| --- | --- |
| Novel method for collecting hippocampal interstitial fluid extracellular vesicles (EVISF) reveals sex-dependent changes in microglial EV proteome in response to Aβ pathology | [https://isevjournals.onlinelibrary.wiley.com/doi/epdf/10.1002/ jev2.12398](https://isevjournals.onlinelibrary.wiley.com/doi/10.1002/jev2.12398) |
| The choroid plexus synergizes with immune cells during neuroinflammation | <https://www.sciencedirect.com/science/article/pii/S0092867424007177> |
| Sex differences in vascular endothelial cells | <https://www.sciencedirect.com/science/article/pii/S0021915023051997> |
| Ex vivo expanded human regulatory T cells modify neuroinflammation in a preclinical model of Alzheimer's disease | <https://link.springer.com/article/10.1186/s40478-022-01447-z> |
| A lysosomal regulatory circuit essential for the development and function of microglia | <https://www.science.org/doi/10.1126/sciadv.abp8321> |
| Elevated TNF-a Leads to Neural Circuit Instability in the Absence of Interferon Regulatory Factor 8 | <https://www.jneurosci.org/content/42/32/6171> |
| Synaptic vulnerability to amyloid-β and tau pathologies differentially disrupts emotional and memory neural circuits | <https://www.nature.com/articles/s41380-025-02901-9> |
| Neurons as Immunomodulators: From Rapid Neural Activity to Prolonged Regulation of Cytokines and Microglia | <https://www.annualreviews.org/content/journals/10.1146/annurev-bioeng-110122-120158> |
| Neuroinflammation in Alzheimer disease | <https://www.nature.com/articles/s41577-024-01104-7> |
| Perivascular cells induce microglial phagocytic states and synaptic engulfment via SPP1 in mouse models of Alzheimer's disease | <https://www.nature.com/articles/s41593-023-01257-z> |
| Microglial TYROBP/DAP12 in Alzheimer's disease: Transduction of physiological and pathological signals across TREM2 | <https://link.springer.com/article/10.1186/s13024-022-00552-w> |
| Type-I-interferon signaling drives microglial dysfunction and senescence in human iPSC models of Down syndrome and Alzheimer's disease | <https://www.sciencedirect.com/science/article/pii/S1934590922002569> |
| Potential role of chitinase-3-like protein 1 (CHI3L1/YKL-40) in neurodegeneration and Alzheimer's disease | <https://alz-journals.onlinelibrary.wiley.com/doi/10.1002/alz.12612> |
| Xenografted human microglia display diverse transcriptomic states in response to Alzheimer's disease-related amyloid-β pathology | <https://www.nature.com/articles/s41593-024-01600-y> |
| Defining blood-induced microglia functions in neurodegeneration through multiomic profiling | <https://www.nature.com/articles/s41590-023-01522-0> |
| Microglial mechanisms drive amyloid-β clearance in immunized patients with Alzheimer's disease | <https://www.nature.com/articles/s41591-025-03574-1> |
| Microglia-synapse engulfment via PtdSer-TREM2 ameliorates neuronal hyperactivity in Alzheimer's disease models | <https://link.springer.com/article/10.15252/embj.2022113246> |
| Identification of a protective microglial state mediated by miR-155 and interferon-γ signaling in a mouse model of Alzheimer's disease | <https://www.nature.com/articles/s41593-023-01355-y> |
| Novel App knock-in mouse model shows key features of amyloid pathology and reveals profound metabolic dysregulation of microglia | <https://link.springer.com/article/10.1186/s13024-022-00547-7> |
| Microglial efferocytosis: Diving into the Alzheimer's disease gene pool | <https://www.sciencedirect.com/science/article/pii/S0896627322009199> |
| Blood-brain barrier leakage and perivascular inflammation in cerebral amyloid angiopathy | <https://cris.maastrichtuniversity.nl/en/publications/blood-brain-barrier-leakage-and-perivascular-inflammation-in-cere/> |
| Targeted BACE-1 inhibition in microglia enhances amyloid clearance and improved cognitive performance | <https://www.science.org/doi/10.1126/sciadv.abo3610> |
| The cytokines interleukin-6 and interferon-α induce distinct microglia phenotypes | <https://link.springer.com/article/10.1186/s12974-022-02441-x> |
| Bacteroidota inhibit microglia clearance of amyloid-beta and promote plaque deposition in Alzheimer's disease mouse models | <https://www.nature.com/articles/s41467-024-47683-w> |
| Microglial repopulation reverses cognitive and synaptic deficits in an Alzheimer's disease model by restoring BDNF signaling | <https://www.sciencedirect.com/science/article/abs/pii/S0889159123002040?via%3Dihub> |
| Single-nucleus RNA-sequencing of autosomal dominant Alzheimer disease and risk variant carriers | <https://www.nature.com/articles/s41467-023-37437-5> |
| Immune checkpoint TIM-3 regulates microglia and Alzheimer's disease | <https://www.nature.com/articles/s41586-025-08852-z> |
| The gain-of-function TREM2-T96K mutation increases risk for Alzheimer's disease by impairing microglial function | <https://www.sciencedirect.com/science/article/pii/S0896627325007457> |
| PICALM Alzheimer's risk allele causes aberrant lipid droplets in microglia | <https://www.nature.com/articles/s41586-025-09486-x> |
| Opposing role of phagocytic receptors MERTK and AXL in Progranulin deficient FTD | <https://www.nature.com/articles/s42003-025-08368-2> |
| Brain-wide microglia replacement using a nonconditioning strategy ameliorates pathology in mouse models of neurological disorders | <https://www.science.org/doi/10.1126/scitranslmed.ads6111> |
| Biophysical mapping of TREM2-ligand interactions reveals shared surfaces for engagement of multiple Alzheimer's disease ligands | <https://link.springer.com/article/10.1186/s13024-024-00795-9> |
| Direct modulation of microglial function by electrical field | <https://www.frontiersin.org/journals/cell-and-developmental-biology/articles/10.3389/fcell.2022.980775/full> |

D) **Glial Metabolism, APOE, and Oligodendrocytes**

| Topic | Link To Paper |
| --- | --- |
| APOE-Targeted Therapeutics for Alzheimer's Disease | <https://www.jneurosci.org/content/45/46/e1388252025> |
| Tau activation of microglial cGAS-IFN reduces MEF2C-mediated cognitive resilience | <https://www.webofscience.com/wos/woscc/full-record/WOS:000975560000007> |
| Concerted type I interferon signaling in microglia and neural cells promotes memory impairment associated with amyloid β plaques | <https://www.sciencedirect.com/science/article/pii/S1074761322001418> |
| APOE4 impairs the microglial response in Alzheimer's disease by inducing TGFβ-mediated checkpoints | <https://www.nature.com/articles/s41590-023-01627-6> |
| Amelioration of Tau and ApoE4-linked glial lipid accumulation and neurodegeneration with an LXR agonist | <https://www.sciencedirect.com/science/article/pii/S0896627323008048> |
| Cell-autonomous effects of APOE4 in restricting microglial response in brain homeostasis and Alzheimer's disease | <https://www.nature.com/articles/s41590-023-01640-9> |
| PLD3 affects axonal spheroids and network defects in Alzheimer's disease | <https://www.nature.com/articles/s41586-022-05491-6> |
| TREM2-independent microgliosis promotes tau-mediated neurodegeneration in the presence of ApoE4 | <https://www.sciencedirect.com/science/article/pii/S0896627322009552> |
| A cell-autonomous role for border-associated macrophages in ApoE4 neurovascular dysfunction and susceptibility to white matter injury | <https://www.nature.com/articles/s41593-024-01757-6> |
| Senescent-like microglia limit remyelination through the senescence associated secretory phenotype | <https://www.nature.com/articles/s41467-025-57632-w> |
| Microglia-astrocyte crosstalk regulates synapse remodeling via Wnt signaling | <https://www.sciencedirect.com/science/article/pii/S009286742500978X> |
| The APOE isoforms differentially shape the transcriptomic and epigenomic landscapes of human microglia xenografted into a mouse model of Alzheimer's disease | <https://www.nature.com/articles/s41467-025-60099-4> |
| APOE genotype determines cell-type-specific pathological landscape of Alzheimer's disease | <https://www.cell.com/neuron/abstract/S0896-6273(25)00135-7> |
| Sex Differences in Longitudinal Tau-PET in Preclinical Alzheimer Disease: A Meta-Analysis | <https://jamanetwork.com/journals/jamaneurology/fullarticle/2830857> |
| Peripheral apoE4 enhances Alzheimer's pathology and impairs cognition by compromising cerebrovascular function | <https://www.nature.com/articles/s41593-022-01127-0> |
| Effect of Race on Prediction of Brain Amyloidosis by Plasma Aβ42/Aβ40, Phosphorylated Tau, and Neurofilament Light | <https://www.neurology.org/doi/10.1212/WNL.0000000000200358> |
| Plasma Aβ42/40 ratio, p-tau181, GFAP, and NfL across the Alzheimer's disease continuum: A cross-sectional and longitudinal study in the AIBL cohort | <https://alz-journals.onlinelibrary.wiley.com/doi/epdf/10.1002/alz.12724> |
| Analysis of changes in intercellular communications in Alzheimer's disease reveals conserved changes in glutamatergic transmission in mice and humans | <https://www.nature.com/articles/s41598-025-10795-4> |
| Confounding factors of Alzheimer's disease plasma biomarkers and their impact on clinical performance | <https://alz-journals.onlinelibrary.wiley.com/doi/epdf/10.1002/alz.12787> |
| Spatially resolved molecular signatures of Lewy body dementia | <https://link.springer.com/article/10.1007/s00401-026-02981-z> |
| Locus coeruleus-entorhinal cortex tract integrity is linked to plasma tau and glial fibrillary acidic protein | <https://alz-journals.onlinelibrary.wiley.com/doi/epdf/10.1002/alz.70915> |
| Aging in mice alters regionally enriched striatal astrocytes | <https://www.nature.com/articles/s41467-025-63429-8> |
| Association of blood-based neurodegenerative biomarkers with cognitive functioning and dementia in India (LASI-DAD) and the United States (HRS) | <https://pmc.ncbi.nlm.nih.gov/articles/PMC12497661/> |
| Frequency and duration of sensory flicker control transcriptional profiles in 5xFAD mice | <https://pubs.aip.org/aip/apb/article/9/3/036102/3351899/Frequency-and-duration-of-sensory-flicker-control> |
| β-Amyloid induces microglial expression of GPC4 and APOE leading to increased neuronal tau pathology and toxicity | <https://link.springer.com/article/10.1186/s13024-025-00883-4> |
| Plasma GFAP for populational enrichment of clinical trials in preclinical Alzheimer's disease | <https://alz-journals.onlinelibrary.wiley.com/doi/epdf/10.1002/alz.70209> |
| Sex differences on tau, astrocytic, and neurodegenerative plasma biomarkers | <https://journals.sagepub.com/doi/10.1177/13872877251329468> |
| Microstructural associations between locus coeruleus, cortical, and subcortical regions are modulated by astrocyte reactivity: a 7T MRI adult lifespan study | [chrome-extension://efaidnbmnnnibpcajpcglclefindmkaj/https://watermark02.silverchair.com/bhae261.pdf](https://pmc.ncbi.nlm.nih.gov/articles/PMC11190376/) |
| Multiomic integration reveals neuronal-extracellular vesicle coordination of gliotic responses in degeneration | <https://onlinelibrary.wiley.com/doi/pdfdirect/10.1002/jev2.12393> |
| Association of plasma biomarkers with cognition, cognitive decline, and daily function across and within neurodegenerative diseases: Results from the Ontario Neurodegenerative Disease Research Initiative | <https://alz-journals.onlinelibrary.wiley.com/doi/epdf/10.1002/alz.13560> |
